# A novel retraining strategy of chest compression skills for infant CPR results in high skill retention for longer

**DOI:** 10.1007/s00431-022-04625-2

**Published:** 2022-09-17

**Authors:** Debora Gugelmin-Almeida, Michael Jones, Carol Clark, Ursula Rolfe, Jonathan Williams

**Affiliations:** 1 Faculty of Health and Social Sciences, Bournemouth University, Bournemouth Gateway Building, St. Paul’s Lane, Bournemouth, BH8 8GP England; 2 Department of Anaesthesiology, Main Theatres, University Hospitals Dorset NHS Foundation Trust - Royal Bournemouth Hospital, Castle Lane East, Bournemouth, BH7 7DW England; 3 Cardiff School of Engineering, Cardiff University, Cardiff, CF23 3AA Wales

**Keywords:** Infant cardiopulmonary resuscitation, Chest compression, Mannequin, Competence-based retraining, Retention of skills, Tailored retraining schedule

## Abstract

Infant cardiopulmonary resuscitation (iCPR) is often poorly performed, predominantly because of ineffective learning, poor retention and decay of skills over time. The aim of this study was to investigate whether an individualized, competence-based approach to simulated iCPR retraining could result in high skill retention of infant chest compressions (iCC) at follow-up. An observational study with 118 healthcare students was conducted over 12 months from November 2019. Participants completed pediatric resuscitation training and a 2-min assessment on an infant mannequin. Participants returned for monthly assessment until iCC competence was achieved. Competence was determined by passing assessments in two consecutive months. After achieving competence, participants returned just at follow-up. For each ‘FAIL’ during assessment, up to six minutes of practice using real-time feedback was completed and the participant returned the following month. This continued until two consecutive monthly ‘PASSES’ were achieved, following which, the participant was deemed competent and returned just at follow-up. Primary outcome was retention of competence at follow-up. Descriptive statistics were used to analyze demographic data. Independent t-test or Mann–Whitney U test were used to analyze the baseline characteristics of those who dropped out compared to those remaining in the study. Differences between groups retaining competence at follow-up were determined using the Fisher exact test. On completion of training, 32 of 118 participants passed the assessment. Of those achieving iCC competence at month 1, 96% retained competence at 9–10 months; of those achieving competence at month 2, 86% demonstrated competence at 8–9 months; of those participants achieving competence at month 3, 67% retained competence at 7–8 months; for those achieving competence at month 4, 80% demonstrated retention at 6–7 months.

*   Conclusion*: Becoming iCC competent after initial training results in high levels of skill retention at follow-up, regardless of how long it takes to achieve competence.

**Table:** 

**What is Known:** *• Infant cardiopulmonary resuscitation (iCPR) is often poorly performed and skills decay within months after training*. *• Regular iCPR skills updates are important, but the optimal retraining interval considering individual training needs has yet to be established*.	
**What is New:** *• Infant chest compression (iCC) competence can be achieved within one to four months after training and once achieved, it can be retained for many months*. *• With skill reinforcement of up to 28 minutes after initial training, 90% of individuals were able to achieve competence in iCC and 86% retained this competence at follow-up*.

**What is Known:**

*• Infant cardiopulmonary resuscitation (iCPR) is often poorly performed and skills decay within months after training*.

*• Regular iCPR skills updates are important, but the optimal retraining interval considering individual training needs has yet to be established*.

**What is New:**

*• Infant chest compression (iCC) competence can be achieved within one to four months after training and once achieved, it can be retained for many months*.

*• With skill reinforcement of up to 28 minutes after initial training, 90% of individuals were able to achieve competence in iCC and 86% retained this competence at follow-up*.

## Introduction

Infant cardiac arrest (up to one year of age) is a major healthcare problem, with high rates of morbidity and mortality [1–3]. High-quality infant cardiopulmonary resuscitation (iCPR) is considered critical to a positive outcome after cardiac arrest [3, 4]. However, it is often poorly performed [5, 6], predominantly because of ineffective learning, poor retention, and decay of skills over time [7, 8].

Current pediatric CPR training is an example of an input-based learning system. The minimum requirements to obtain certification depend on subjective opinion that the instructor makes about an individual’s performance against some pre-set criteria [9], or via a self-assessment tool [10], without a quantitative assessment of performance. It has been suggested that this model does not result in CPR competence and often leads to poor retention, with skills declining within weeks to months after training [7, 8, 11–15]. The poor retention of skills can potentially affect performance and patient outcomes, raising the question as to why the current training strategy of yearly updates is thought to be optimal for maintaining the quality of CPR skills.

To address skill decay, researchers have investigated different models of distributed retraining schedules, suggesting that monthly retraining generates the best retention [15, 16]. However, this is associated with dropout rates of up to 50% [15] and significant increased costs of repeated training, which may affect the viability of rolling out the training into practice. When attempting to avoid skill decay, regular updates are important [8], but the optimal balance between CPR skills retraining and sustainability has yet to be established.

Previous studies have explored retraining at predefined intervals (i.e., every month); however, this fails to take into consideration the degree of learning already achieved. It might be suggested that those struggling to acquire iCPR competence are likely to require more input than those who have previously mastered the skills. Therefore, a randomized controlled trial design exploring different retraining schedules at pre-set intervals fails to take into consideration the needs of the individual. For this reason, the present study adopted a competence-based approach design to iCC retraining. This type of training model is based on the ‘mastery learning strategy,’ where learners have the opportunity to practice key skills, receive feedback and improve performance until mastery is achieved [17]. Retraining is then individualized and is based on the quality of their performance in achieving a predefined level of competence [17]. Using the mastery learning strategy to achieve a competence-based approach to iCC may lead to long-term retention of skills, delivery of timely high quality iCC, and subsequent cost savings due to reduced retraining. Despite the importance of ventilation during iCPR [3, 4], this study specifically focused on chest compression skills during iCPR.

The aims of this study were: (i) to determine the amount of input needed to achieve iCC competence, based on achieving four internationally recommended quality measures: chest compression depth, chest compression rate, complete chest recoil and compression duty cycle (the portion of time spent in compression); and (ii) to determine if the acquired competence is retained over time.

This is the first time an individualized design has been explored, to establish whether an outcome-based approach (competence) to a retraining schedule of iCC skills could result in skill retention, while minimizing the potential for dropouts.

## Methods

A prospective, observational study was conducted over 12 months within a simulated environment. University Research Ethics Committee approval was obtained (reference ID:27,402). Following explanation of the experimental procedures, written informed consent was obtained from all participants before commencing the study. To create a demographic profile of the sample, data related to age, sex, height, weight and self-declared physical issues, that might compromise performance, were collected.

## Participants

A sample size of 72 was calculated based on Anderson et al. [15] at 12-month completion, with alpha at 0.05 and beta at 0.8. However, due to an anticipated high dropout rate (based on previous studies) [15, 16], a convenience sample of 118 university healthcare students was recruited (September–November 2019). Inclusion criteria: (a) university enrolment; (b) not in their final year of study; (c) no previous pediatric CPR training. Exclusion criteria: any musculoskeletal condition requiring medical intervention or an inability to physically perform iCPR.

## Equipment

Equipment comprised of an ALS Baby mannequin representing a 5 kg, three-month-old infant (Laerdal® Medical, Stavanger, Norway), modified in a previous study to allow chest compression to a physiological 56 mm depth [18]. The mannequin was attached to a wooden board and during data collection, was placed on a hard floor to eliminate the damping effect of mattress compressibility [19]. The mannequin was instrumented with two accelerometers, one at the lower third of the sternum, the other on the board, acting as a differential for the surface. The mannequin was connected to a PC via a data acquisition unit, where acceleration data were converted into displacement [20].

A bespoke MATLAB algorithm generated four metrics of chest compression during iCPR performance: chest-compression-depth (CCD), chest compression-rate (CCR), residual leaning (RL) and duty-cycle (ratio of compression:release) (DC) (see Table 1). Although participants did not receive real-time-feedback during assessment, Labview software, developed and validated for a previous study [18], provided real-time feedback if needed during practice between assessments. Therefore, the algorithm was able to assess the quality of chest compressions during iCPR and determine if the performance met the target ranges described in Table 1.

**Table 1:** Definitions of the four iCC skill elements; chest compression rate, chest compression depth, residual leaning and duty cycle. The target ranges and justifications are provided

Metric	Definition	Target range	Target range justification
Chest compression rate	The number of compressions per minute	100–120 min^−1^	Based on European Resuscitation Council (ERC) and American Heart Association (AHA) guidelines [3, 4]
Chest compression depth	The maximum relative displacement between the two accelerometers during each compression	> 35–45 mm	ERC and AHA guidelines recommend a compression depth of at least one-third the external anterior–posterior chest diameter for an infant (approximately 40 mm) [3, 4]. Upper threshold was selected based on the hypothesis that a residual internal anterior–posterior chest depth of < 10 mm may potentially cause intra-thoracic trauma [21]. Lower threshold was selected based on this mannequin’s specification (to achieve one-third the external anterior–posterior chest diameter)
Residual leaning	The incomplete release from the chest wall after each compression	< 2.5 kg	Inadequate recoil (> 2.5 kg) causes high intrathoracic and right atrial pressure, reducing coronary perfusion, venous return to the heart, and blood flow generated by the next compression [22]
Compression duty cycle	The ratio of time taken for compression relative to release	30–50%	Based on Resuscitation Council UK (RCUK) guidelines [23]. The lower threshold was selected based upon the hypothesis that a shorter duty cycle provides significant superior myocardial and cerebral perfusion in infant swine model [24]

### Intervention

The intervention was comprised of three aspects:

1. Training: all participants received the four-stage approach ‘Pediatric Basic Life Support (BLS)’ education package [9], provided by a RCUK qualified instructor. This training was completed in small groups covering aspects such as choking, recovery position, theoretical background in CPR, chain of survival, rescue breaths, chest compressions, different techniques relevant to different ages (e.g., two-thumb, two-finger, one hand, two hands), paediatric modifications, retrieving and using automated external defibrillator, etc. After two-hour training, participants practiced the two-finger compression technique for 30 min on the mannequin, using a compression:ventilation ratio of 30:2 (aligned with resuscitation guidelines for single BLS rescuers with no duty to respond to pediatric cardiac arrest) [23].
2. Assessment: all participants individually completed simulated iCPR on the mannequin for two minutes and performance data based on CCR, CCD, RL and DC were captured (this was called the ‘assessment’). Instruction was provided when to start and stop but no further instruction or feedback were provided during assessment so that performance was not influenced. iCC quality was determined using a MATLAB algorithm, to establish if a “PASS” was achieved—defined as achieving an average of compressions within the target ranges for each metric, as shown in Table 1. These metrics have been demonstrated to provide high repeated measures reliability [25]. All performance metrics (CCR, CCD, RL and DC) needed to be achieved, concurrently, to be considered a “PASS”.
3. Practice with real-time feedback: this step was only completed if a participant “FAILED” an assessment. Participants practiced iCPR for 1 min using real-time feedback, after which, an evaluation of performance was completed for 2-min. If the participant “FAILED” again, another block of 1-min practice plus 2-min re-evaluation was completed. No further practice was permitted. The use of real-time feedback during CPR training has been demonstrated to improve CPR quality [26] and the feedback tool used in this study has been described previously [20].

### Participant flow through the study

**Fig. 1:**
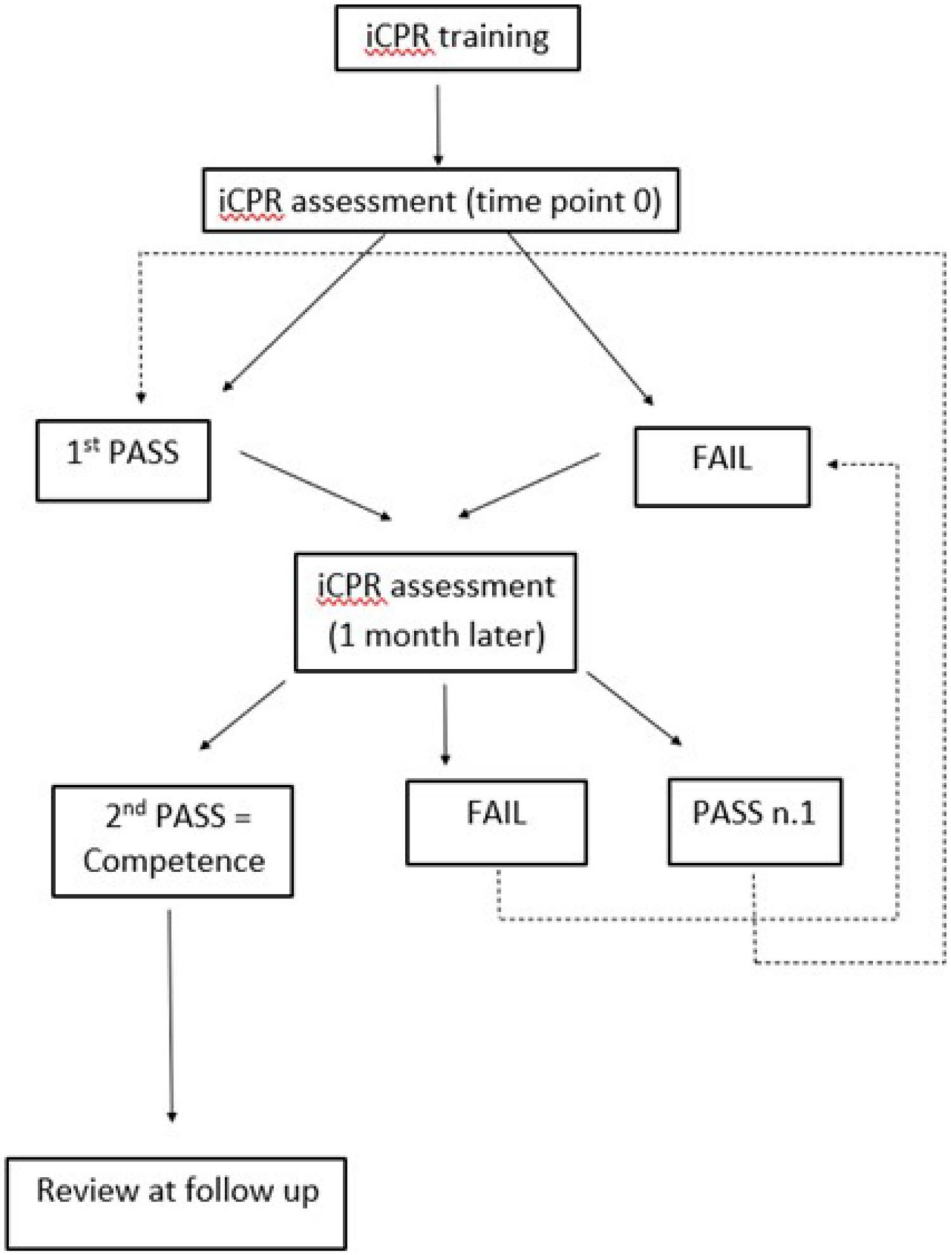
demonstrating participant’ flow through the 12-month study following initial iCPR training

The flow of participants through the 12-month study was determined by the individual performance during iCC assessments according to the following protocol (see Fig. 1):

### Time point 0 (immediately after training)

If a ‘PASS’ was achieved, the participant returned the following month for a repeat assessment. However, if the participant ‘FAILED,’ practice with real-time feedback was completed (as explained in step 3 of the ‘intervention’ above). The participant then returned the following month for a repeat assessment.

### Time point 1 (one month later)

Participants with a previous ‘PASS’ were reassessed. If another ‘PASS’ was achieved, they were declared iCC competent and returned just at follow-up. However, if they ‘FAILED’ at this time, the same process for ‘FAILED’ participants at time point 0 was followed.

Participants with a previous ‘FAIL’ at time point 0 were also reassessed. If they ‘FAILED’ again, the same process for ‘FAILED’ participants at time point 0 was repeated. If instead, a ‘PASS’ was achieved at time point 1, the participant returned the following month for another assessment.

Therefore, participants returned monthly until a ‘PASS’ was achieved for two consecutive months, after which they were declared iCC competent and returned just at follow-up. If the participant ‘FAILED’ any assessment, even if they had a previous ‘PASS’, they were required to return monthly until two consecutive ‘PASSES’ were achieved. Once achieved, they were declared iCC competent and were asked to return just at follow-up.

The overarching aim was to achieve iCC competence (declared as two consecutive monthly ‘PASSES’) and participants were grouped for analysis according to when they achieved competence.

This study was completed over a 12-month period, therefore, the follow-up period varied between six to 10 months, due to the staggered nature of when competence was achieved. At follow-up, a repeat iCC assessment was completed. No prior practice was permitted. The choice of two consecutive monthly “PASSES” was based on a previous study, which concluded that while monthly CPR re-exposure is effective for retention at follow-up, two months may be sufficient [16].

### Outcome measures

Primary outcome: retention of iCC competence, defined as achieving a “PASS” during iCPR assessment at follow-up.

Secondary outcome: the length of time taken for iCC competence to be achieved.

### Statistical analysis

Descriptive statistics were used to analyze demographic data. Mean (SD) values were used to report data with a normal distribution and median [IQR] values were used when the assumption of normality was not met via the Skewness, Kurtosis and Shapiro–Wilk assessment.

The baseline characteristics of those who dropped out were compared to the baseline of those remaining in the study, using an independent t-test or Mann–Whitney U test, depending on the distribution of the variable.

The differences between groups retaining competence at follow-up were determined using the Fisher exact test.

SPSS Statistics version 25 (IBM Corp., Armonk, NY, USA) was used for statistical calculations.

## Results

### Study population

A total of 118 participants were enrolled in the study including 84 females (71%). The median [IQR] age was 23 [11] years, weight was 68 [17] kg and mean (SD) height was 170 (9) cm.

### Trajectory of participants

A total of 87 participants (74%) completed the 12-month study. Drop-outs were due to: Covid-19 (15); long commutes (4); work or placement (5) or non-contactable (7). Participants who completed the study were not statistically different from those who dropped out with respect to demographics or baseline iCC performance at time point 0, as demonstrated in Table 2.

**Table 2:** Comparison between participants who completed the study and those lost at follow-up

	Completed	Leavers	P value
Number	87	31	
Age (years)	23 [11]^β^	25 [11]^β^	0.75^a^
Weight (kg)	68 [16]^β^	69 [21]^β^	0.66^a^
Height (cm)	170.1 (8.6)	168.2 (11.0)	0.33
Average chest compression rate at baseline (cpm)	101.3 (14.6)	101.0 (16.8)	0.92
Average chest compression depth at baseline (mm)	41.7 [4]^β^	43.1 [4]^β^	0.26^a^
Average residual leaning at baseline (kg)	3.0 (0.8)	3.0 (1.2)	0.75
Average duty cycle at baseline (%)	45.7 [8.0]^β^	47.8 [8.1]^β^	0.60^a^

*Cpm* compression per minute

^a^Mann–Whitney U test for non-parametric

^β^Median and interquartile range

### Initial assessment results following completion of iCPR training (time point 0)

A total of 118 participants undertook iCPR training and were assessed immediately after training (time point 0). Of the 118 participants, a total of 32 (27%) ‘PASSED’ on the first attempt and were asked to return the following month for another assessment. The 86 (73%) participants who failed the assessment, used real-time feedback for reinforcement of skills and were also asked to return the following month for another assessment.

Group 1: Participants achieving iCC competence one month after training (time point 1).

One month after the initial training, the 32 participants who had ‘PASSED’ at time point 0 were invited to reassessment (time point 1). One dropped out, leaving a total of 31. Of those, 28 (90%) achieved iCC competence by passing the assessment at time point 1.

**Fig. 2:**
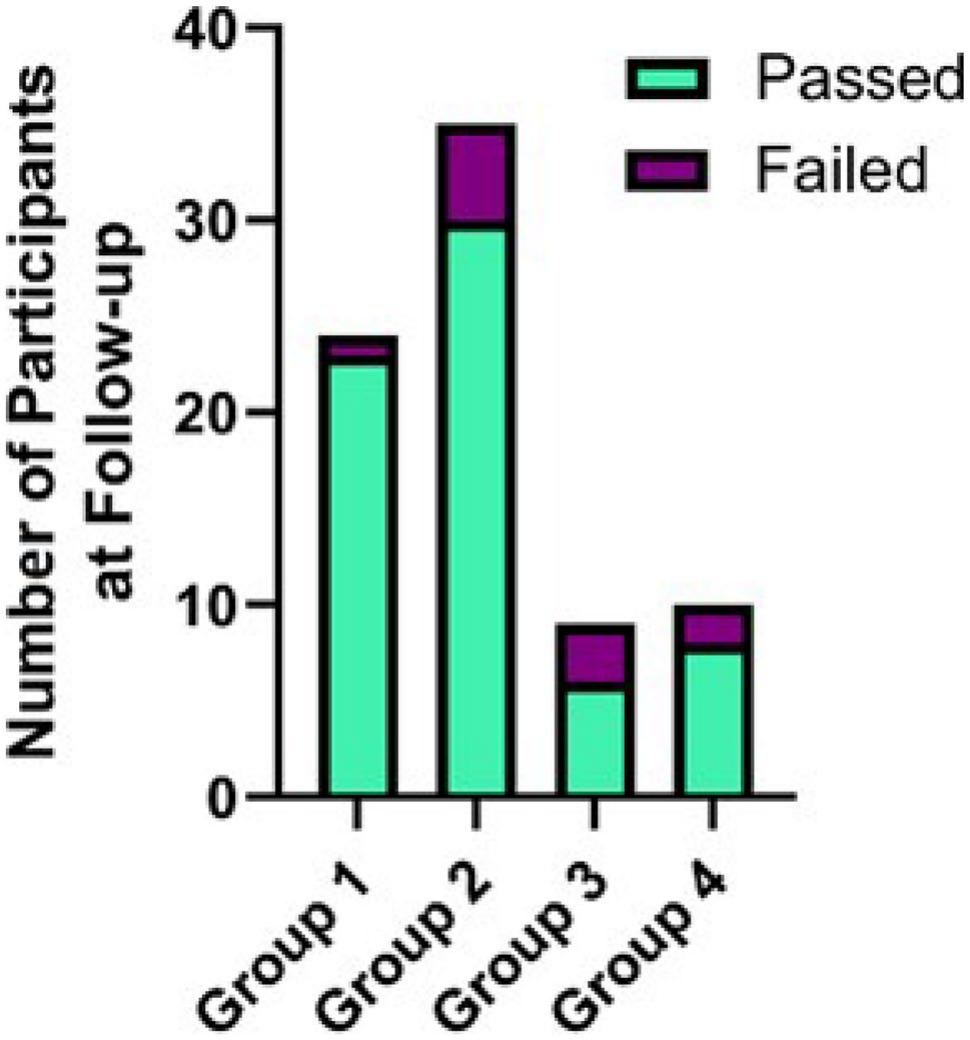
demonstrating the number of participants from each group who retained iCC competence at follow-up by passing the assessment. Group 1 had 9–10 months follow-up, group 2 had 8–9 months follow-up, group 3 had 7–8 months follow-up and group 4 had 6–7 months follow-up. The number of participants who hadn’t retained competence at follow-up is reported

At 9–10 months follow-up, a further four participants left the study, leaving a total of 24 participants in this group. Of those remaining, 23 (96%) demonstrated retention of competence by passing the assessment at follow-up (see Fig. 2).

Group 2: Participants achieving iCC competence two months after training (time point 2).

Two months after the initial training (time point 2), the 44 who failed at time point 0 but then passed at time point 1 were invited to reassessment. Of those, 41 (93%) achieved competence at time point 2 by passing the assessment.

At 8–9 months follow-up, of the 41 participants who achieved competence at time point 2, six left the study, leaving a total of 35 participants in this group. Of those remaining, 30 (86%) demonstrated retention of competence by achieving a pass at follow-up assessment (see Fig. 2).

Group 3: Participants achieving competence three months after iCPR training (time point 3).

Three months after the initial training (time point 3), the 12 participants who failed at time point 1 but then passed at time point 2 were invited to reassessment. All (100%) achieved competence at time point 3 by passing the assessment.

At 7–8 months follow-up, of the 12 participants who achieved competence at time point 3, three left the study, leaving a total of nine participants in this group. Of those remaining, six (67%) demonstrated retention of competence with a pass at follow-up assessment (see Fig. 2).

Group 4: Participants achieving competence four months after iCPR training (time point 4).

Four months after the initial training (time point 4), the 11 participants who failed at time point 2 but then passed at time point 3, were invited to reassessment. All (100%) achieved competence at time point 4 by passing the assessment.

At 6–7 months follow-up, of the 11 participants who achieved competence, one left the study, leaving 10 in this group. Eight (80%) demonstrated retention of competence with a pass at follow-up assessment (see Fig. 2).

Application of the Fisher exact test demonstrated no significant difference between groups for skill retention at follow-up (p = 0.13), indicating that once competence was achieved, the likelihood of retaining the skills at follow-up was no different across the groups.

## Discussion

The aims of this study were to establish the amount of input required to achieve iCC competence and to determine if the acquired competence was retained at follow-up. This was achieved through an individualized ‘competence-based retraining schedule,’ with input based on the performance and retention of iCC skills and therefore, offers a unique approach to iCPR retraining. To the authors’ knowledge, this is the first study to explore a tailored approach to iCC retraining based on individual performance. It was demonstrated that within four months of the initial training, 90% of participants had achieved iCC competence and the likelihood of the participants retaining the skills for up to 10 months was as high as 96%.

The baseline results at time point 0 indicate a similarity to previous pediatric CPR research [5, 6, 11–16, 27, 28], in that, although our participants took part in a two-hour pediatric BLS training, only 27% performed adequately (as defined in our study) immediately after training. This suggests the current input training model struggles to adequately prepare individuals to be competent and brings into question the efficacy of this model of training. Current pediatric BLS training varies significantly but commonly involves attendance at a two-hour instructor-led session every one or two years [7, 8], often without measurement of competence. The minimum requirements to obtain certification are normally visually assessed by the instructor or via a self-assessment tool [9, 10]. Although some participants may achieve the minimum requirements, previous researchers have established that performance immediately after training is consistently poor [5–8, 11–16, 27] and the skills may deteriorate within as little as one month after training [15]. It might be postulated that this traditional strategy of pediatric CPR training should, therefore, be reviewed for effectiveness.

Previous resuscitation studies have concluded that increasing the frequency of retraining may result in improvements in CPR skill retention and clinical outcomes after a cardiac arrest [7, 8]. There are, however, conflicting opinions regarding the frequency of skills re-exposure. Studies exploring the use of distributed practice or low-dose exposure at higher frequency agree that retention of CPR skills is enhanced by short period training sessions [11–14]. Similarly, evidence about mastery learning, where learners practice key skills, receive directed feedback and improve performance until mastery is attained, demonstrates that repetition paired with feedback enables learners to consistently demonstrate a predefined level of competence for a specific skill [17].

Nevertheless, the evidence as to what constitutes an ideal timeframe of repetition is inconclusive. Some studies support weekly [28] or monthly retraining [13, 15, 16], while others suggest that retraining every six [14, 29] or 12 months [30] is sufficient. In practice, the optimal reinforcement schedule is highly likely to vary depending on an individual, and this was demonstrated within the present study. Some participants were able to achieve iCC competence quickly and retain it until follow-up, while others needed more exposure to facilitate learning. Interestingly, despite the varied time frame required to achieve iCC competence, this had little effect on the likelihood of retaining competence at follow-up. This suggests that, once individuals become competent, they can retain competence for at least the duration of follow-up explored in this study. This is in contrast with other studies, which demonstrate significant decay of skills after one to six months [11–16, 28], perhaps intimating that competence was not achieved after training. Therefore, a more nuanced approach to iCPR retraining, based on an individuals’ performance is recommended, in an attempt to maximize skill retention, improve performance and potentially enhance patient outcome.

The results from the current study demonstrate higher retention of iCC skills, when compared to previous studies. No ‘usual training’ control group was included in the current study as the literature for retention following a single point of training demonstrates unequivocally that retention of skills is poor, at just 15–20% of individuals at 12-month follow-up [13, 15]. Distributed monthly practice has demonstrated retention of skills, with 54%–58% of individuals achieving high quality CPR at 12-month follow-up [13, 15]. However, the current study demonstrated retention rates ranging from 67 to 96% demonstrating the efficacy of a tailored approach to learning.

The tailored approach adopted in the current study relied on the 2-h training (time point 0) plus additional practice using real-time feedback until competence was achieved. Cost was not considered in this study, nevertheless, additional practice is likely to result in greater costs, mainly due to individuals spending time absent from clinical areas. It was noted, however, that all individuals achieved competence within four months, so the maximum amount of additional input to achieve competence following this retraining model was up to 28 min. Therefore, for a maximal additional cost of up to 28 min, individuals were able to not only achieve competence in iCC, but also retain this competence at follow-up, potentially reducing training costs overall. It should be acknowledged that this study focused on the development and retention of iCC skills, and this may not reflect training times for the full cycle of iCPR, which includes rescue breaths.

The limitations of this study include: iCC assessments were individually performed in a simulated environment and for two minutes only. It must be acknowledged that real life CPR will be more stressful and may continue for considerably longer. Therefore, participants were not exposed to stress, distractions or fatigue that may occur during CPR, limiting the transferability of the results to real life performances. A convenience sample of participants was recruited from a single institution, potentially limiting the generalizability of our results to a wider population. A mannequin was used to assess the quality of iCC skills. Although several studies investigating CPR performance use mannequins, it is recognized that chest compliance may not be entirely biofidelic. Ventilation and hands-off time are extremely important elements of iCPR. However, these were not investigated in this present study as the focus was chest compression quality and retention. This study did have a drop out of 26%. This is typical of this type of research, and future studies could investigate methods to minimize drop out.

Despite its limitations, this study demonstrated that achieving iCC competence after training results in high levels of skill retention at follow-up, regardless of how long it takes to achieve competence.

## Abbreviations

AHA: American Heart Association
ALS: Advanced life support
BLS: Basic life support
CCD: Chest compression depth
CCR: Chest compression rate
CPR: Cardiopulmonary resuscitation
DC: Duty cycle
ERC: European Resuscitation Council
iCC: Infant chest compression
iCPR: Infant cardiopulmonary resuscitation
IQR: Interquartile range
RCUK: Resuscitation Council UK
RL: Residual leaning
SD: Standard deviation
